# Improvement of Contused Spinal Cord in Rats by Cholinergic-like Neuron Therapy

**DOI:** 10.5812/ircmj.7653

**Published:** 2013-02-05

**Authors:** Majid Naghdi, Taki Tiraihi, Seyed Alireza Mesbah-Namin, Jalil Arabkharadmand, Hadi Kazemi, Taher Taheri

**Affiliations:** 1 Department of Anatomical Sciences, Faculty of Medical Sciences, Tarbiat Modares University, Tehran, IR Iran; 2 Department of Anatomical Sciences, Faculty of Medical Sciences, Tarbiat Modares University; Shefa Neuroscience Research Center, Khatam Al-anbia Hospital, Tehran, IR Iran; 3 Department of Clinical Biochemistry, Faculty of Medical Sciences, Tarbiat Modares University, Tehran, IR Iran; 4 Shefa Neuroscience Research Center, Khatam Al-anbia Hospital, Tehran, IR Iran

**Keywords:** Spinal Cord Injuries, Contusions, Cholinergic Neurons, Tissue Therapy

## Abstract

**Background:**

Disability in spinal cord injury is an important medical problem, and cell transplantation is considered as an option for the treatment.

**Objectives:**

The purpose of this study is to use bone marrow stromal cells (BMSCs) derived cholinergic neuron-like cells (CNL) in order to ameliorate the contusion model of spinal cord injury in rats.

**Materials and Methods:**

The CNLs were produced by pre inducing BMSCs with β-mercaptoethanol (BME) followed by inducing with nerve growth factor (NGF). The cells were immunoreactive to neurofilament 200, NeuN, synaptophysin, synapsin, microtubule associated protein-2 and choline acetyl transferase (ChAT). The CNL were transplanted in contused rats (CR), which were sacrificed after 12 weeks.

**Results:**

The results showed that BBB test showed an improvement in the CR, while the quantitative analysis showed that the improvement rate was higher in the rats treated with CNL than those treated with BMSCs only or the untreated animals, similar results were noticed in the improvement index. Immunohistochemical analysis of the tissue section prepared from the CR showed that the transplanted cells were engrafted and integrated in the traumatized spinal cord. The morphometric analysis showed that the volume density of the cavity in the CNL treated rats was significantly lower than that of the untreated ones, while the spinal tissue regeneration index was significantly higher.

**Conclusions:**

The conclusion of the study is that CNL can improve the injured spinal cord.

## 1. Background

McDonald et al. transplanted neural phenotype derived from mouse embryonic stem cells (ESCs) into rats with spinal cord injury, which improved their locomotors activities (1). However, Henon suggested using adult stem cells as an alternative source for cell therapy because of the ethical problems in the clinical use of ESCs (2). Newman et al. mentioned that the hematopoietic system derived cells such as bone marrow stromal cells (BMSCs) and umbilical cord cells could be an alternative source for the treatment of central nervous system injury models because of their ability to differentiate into neuron-like cells and to engraft the traumatized tissues with functional recovery (3). Other investigators agreed with them (4-8). Moreover, Romano emphasized the safety concerns of using ESCs in clinics and suggested the use of adult stem cells for transplantation (9). Li et al. (10) reported the adverse effects of ESC transplantation in different models of neurodegeneration including tumor growth and immune rejections, similar concerns were addressed by Coutts and Keirstead (11). Chopp et al. transplanted BMSCs in an animal model of spinal cord injury, which improved the behavioral test results (12). Hofstetter et al. justified the use of BMSCs for their accessibility and expandability (13). One of the major advantages of BMSCs transplantation in spinal cord injury is its autologous feature (14). Intraspinal transplantation of undifferentiated BMSCs was reported to express neuron, astrocyte (15) and oligodendrocyte markers (16). While Verdú et al. evaluated the injured spinal cord by measuring the size of the cystic cavities, which was larger in the untreated animals than those treated with olfactory ensheathing cells transplant (17). Zurita and Vaquero (18) confirmed that BMSCs could improve chronic paraplegia and reduce the size of spinal cord injury cavitation (14, 19), where the transplanted cells integrated in the injured spinal cord tissues (20). In the transplantation of primed human NSCs in a contusion model of rat spinal cord, the cells differentiated into cholinergic neurons, which improved the locomotion of the injured rats, however, the improvement depended on several variables such as post injury transplantation timing, transplantation site and the survival of the differentiated cells (21). Moreover, partial improvement of the atomized Moto neurons in the newborn was also documented (22), Hou et al. used self-assembling peptide seeded with Moto neurons differentiated from fetal neural stem cells and transplanted in injured spinal cords of rats, which resulted in partial functional recovery (23). One of the functions of the cholinergic neurons in the spinal cord is motor activities (24).

## 2. Objectives 

In this study, the improvement of locomotion in the contused rats transplanted with cholinergic neurons derived from autologous BMSCs has been evaluated by using quantitative methods.

## 3. Materials and Methods

The trans differentiation of the cholinergic neuron-like cells from the BMSCs was done according to Naghdi et al. (25), briefly, the BMSCs were prepared from 6-8-week old Sprague-Dawely rats by removing and cutting the ends of the femurs and the tibias, they were flashed out with 5 ml of αMEM medium (Gibco) supplemented with 10% FBS (Gibco) and cultured in the same medium (supplemented with 10% FBS, penicillin and streptomycin) for 24 hrs. The adherent cells were used for differentiating the BMSCs after the fifth passage, and the features of the BMSCs were evaluated using anti-fibronectin, anti-CD 45, anti-CD 44 and Oct-4 expression using RT-PCR. The differentiation protocol is consisted of a pre induction stage using β-mercaptoethanol for 24 hours (BME: 1mM) followed by an induction stage using the nerve growth factor for 2, 4 and 6 days (3, 5 and 7 days from the beginning of the differentiation protocol) (NGF: 100 ng/ml). A cocktail of antibodies (primary antibodies) against neuronal markers was used in order to evaluate the differentiation by immunocytochemistry, including nestin, NF-68, NF-200, MAP2, NeuN, synaptophysin and the cholinergic neuron marker (choline acetyl transference: ChAT), which was also detected by immunocytochemistry. The BMSCs derived cholinergic neuron-like cells (CNL) used for the in vivo study were labeled with bromodeoxyuridine (BrdU: 0.1 mM, Sigma) by adding BrdU into the culture 72 hours before the pre induction. The female Sprague-Dawley rats (230–250 g) were purchased from Razi Institute, Tehran, Iran. The animals were divided into five groups: sham operated (S), contused without treatment (C), placebo (P: contused animals treated with 9 µL normal saline only, which was used as the vehicle and was injected intraspinally at the epicenter, rostral and caudal of the impact site), the BMSCs treated group (B: 300,000 BMSCs in vehicle injected as above) and the cholinergic trans differentiated neurons from the BMSCs (N: 300,000 CNL in vehicle injected as above). The contusion was carried out by using the New York Weight drop device (NYW) (26). Ketamine (80mg/kg) and xylazine (10mg/kg) were used in order to anesthetize the animals, which were laminectomies at T13, and a 10 gm weight rod was dropped from a height of 2.5 cm onto the exposed spinal cord, then the muscles and skin were sutured over the laminectomies vertebra. Postoperative care was done using Ringer lactate (subcutaneous: 5 ml) and ceftazoline (50 mg/kg) twice a day for 3 days, and Tramadol (20 mg) for 2 days. The animals were maintained for 12 weeks and an open field test was done on all of them (in the experimental and control groups) according to Basso-Beattie-Bresnahan scale (BBB scale). They were subjected to pre surgical training for 10 days, then BBB test was done on the day of the surgery (day 0) and days 4, 7, 8, 11, 14, 21, 28, 35, 42, 49, 56, 63, 70, 77 and 84. The data were analyzed by non-linear regression using the Logistic model, the “c” coefficient was considered as the improvement rate (26, 27). The immunostaining was done on the cultured cells as follows: they were washed with phosphate buffer saline (PBS), fixed in acetone, rewashed with PBS, permeated with 0.3% triton X-100, blocked with 10% normal goat serum and incubated with a primary antibody, that was followed by FITC conjugated secondary antibody. The immunolabeled cells (200 cells) were counted at 200 X from random fields on the immunostained coverslips. Double labeling immunohistochemistry was done on the spinal tissues cut with cryostat from the groups, then they were immunolabeled with anti-BrdU primary antibody, which was followed by incubation with Rhoda mine conjugated secondary antibody. The tissues were then double labeled with anti-ChAT primary antibody and incubated with FITC conjugated secondary antibody. The numerical density per area, the mean of the numerical density of the transplanted BMSCs per area and the mean of the numerical density of the transplanted trans differentiated cholinergic neuron-like cells per area were evaluated. Similar groups were perfused with paraformaldehyde, processed for paraffin, cut and stained with hematoxylin and eosin. The mean of the volume density of the spinal cavity at the epicenter, rostral and caudal of the impact site were calculated. The spinal tissue regeneration index (STRI) was calculated as follows:

( in treated animals - in untreated animals)

STRI = 1 - V̅ v_cavity_ in treated animals - V̅ v_cavity_ in untreated animals / V̅ v_cavity_ in untreated animals

in untreated animals

The data were statistically analyzed by using SPSS package (www.spss.com) and the normality of the data was evaluated by the one-sample Kolmogorov-Smirnov test (SK test) and the one way analysis of variance (ANOVA). Turkey’s test post Hoc was used for analyzing the results.

## 4. Results

**Figure 1:**
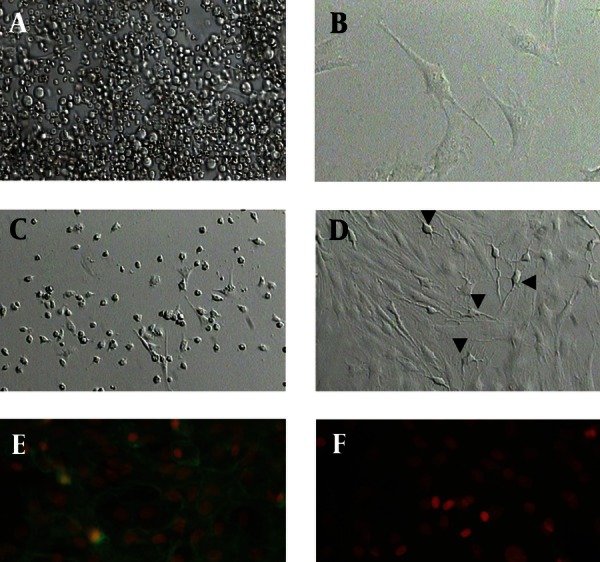
Phase Contrast Photomicrographs from Bone Marrow Stromal Cells( BMSCs) with Different Preparations (A-D) and Immunostaining of BMSCs with Different Markers in order to Characterize These Cells (E and F)

**Figure 2:**
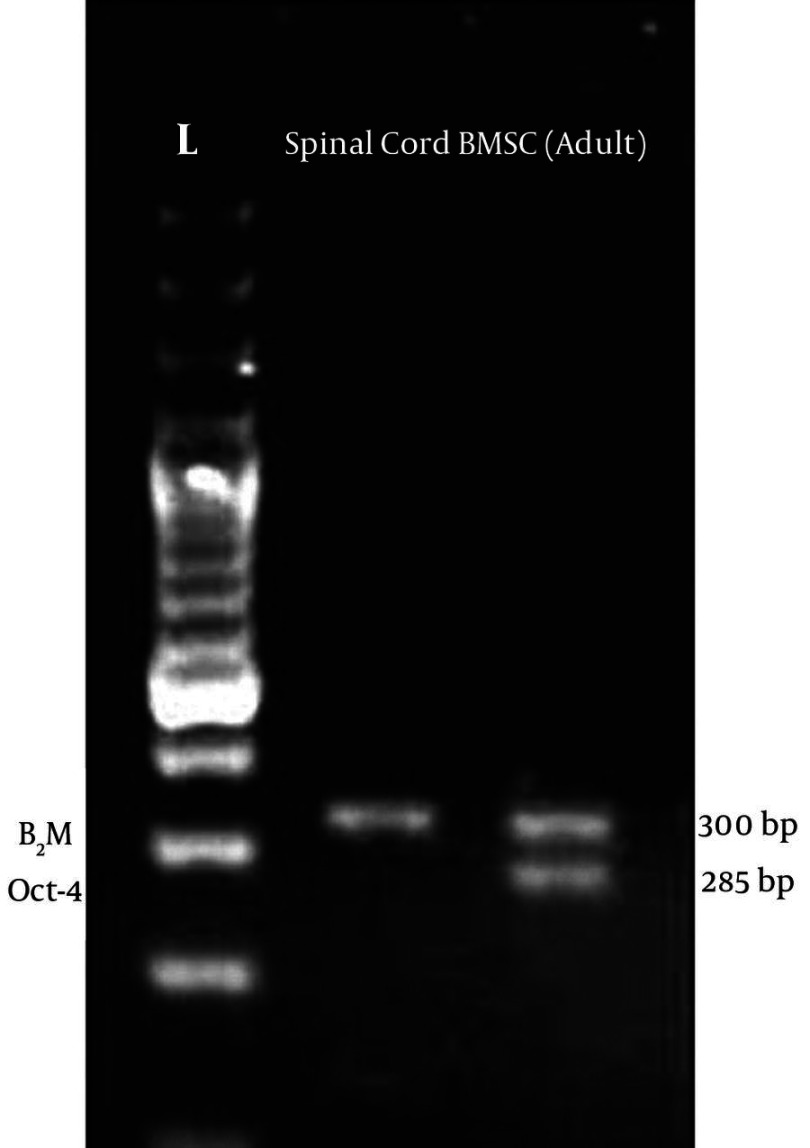
Electrophogram of Oct-4 in the Adult Spinal Cord (a differentiated tissue) Used as Negative Control

**Figure 3:**
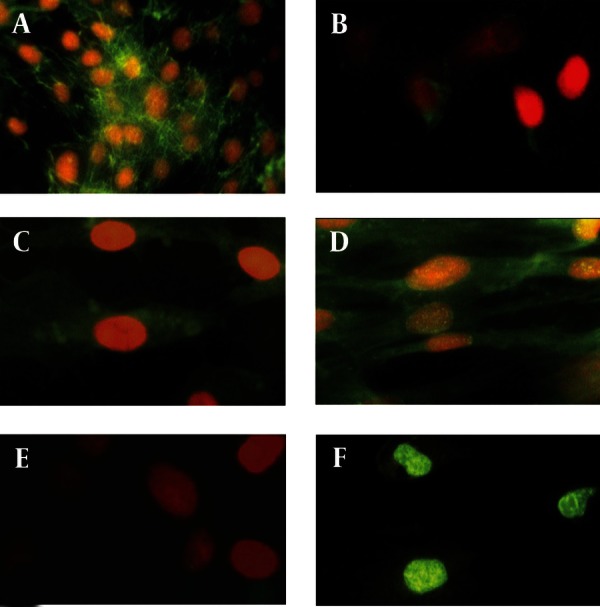
Immunostaining of Bone Marrow Stromal Cells ( BMSCs) With Anti-Fibronectin Antibody A), and Pre induced and Induced BMSCs with Anti-neurofilament (NF) Antibodies (NF-200: C and E; and NF-68: B and D) and Induced BMSCs Labeled with BrdU (F)

**Figure 4:**
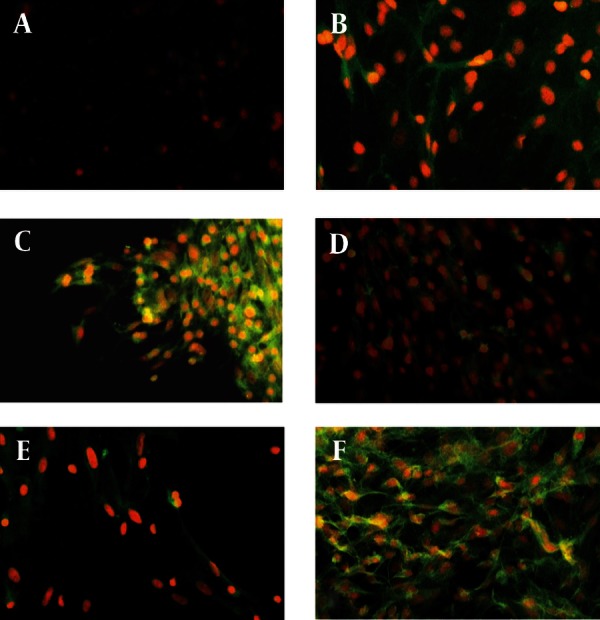
Immunostaining of Bone Marrow Stromal Cells (BMSCs) with Anti-Synaptophysin (A and C), Anti-MAP2 (B and E), and Anti-choline Acetyl Transferase (ChAT: D and F)

**Figure 5:**
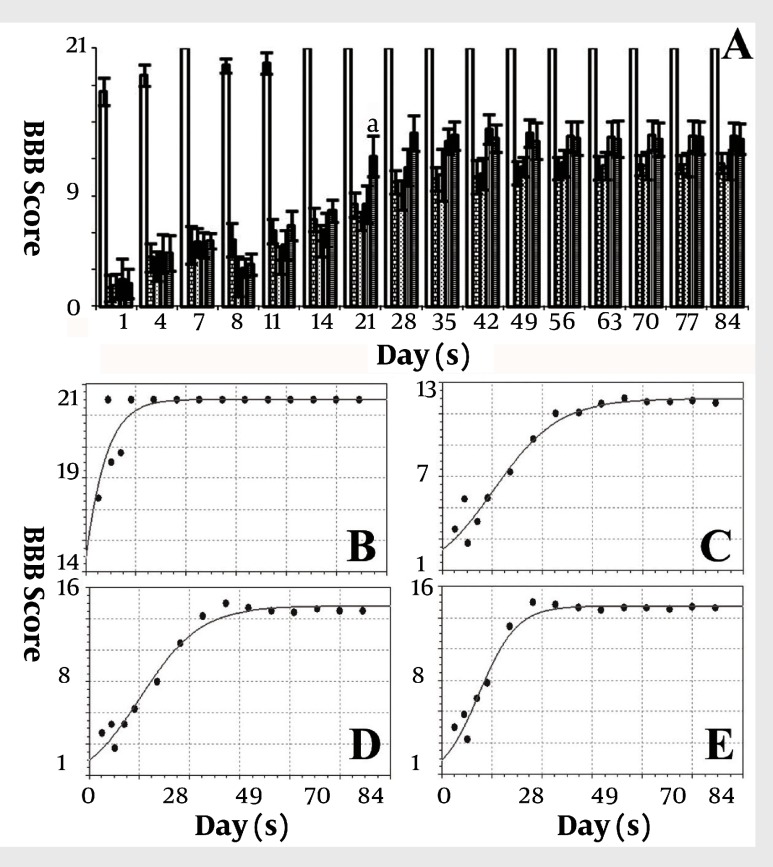
A represents a histogram of BBB test for the sham-operated (solid white), the negative control group injected without treatment (brick pattern), the negative control group injected with normal saline (dotted pattern), the animal group treated with bone marrow stromal cells (vertical lines pattern) and the animal group treated with cholinergic neuron-like cells (horizontal lines pattern)

**Figure 6:**
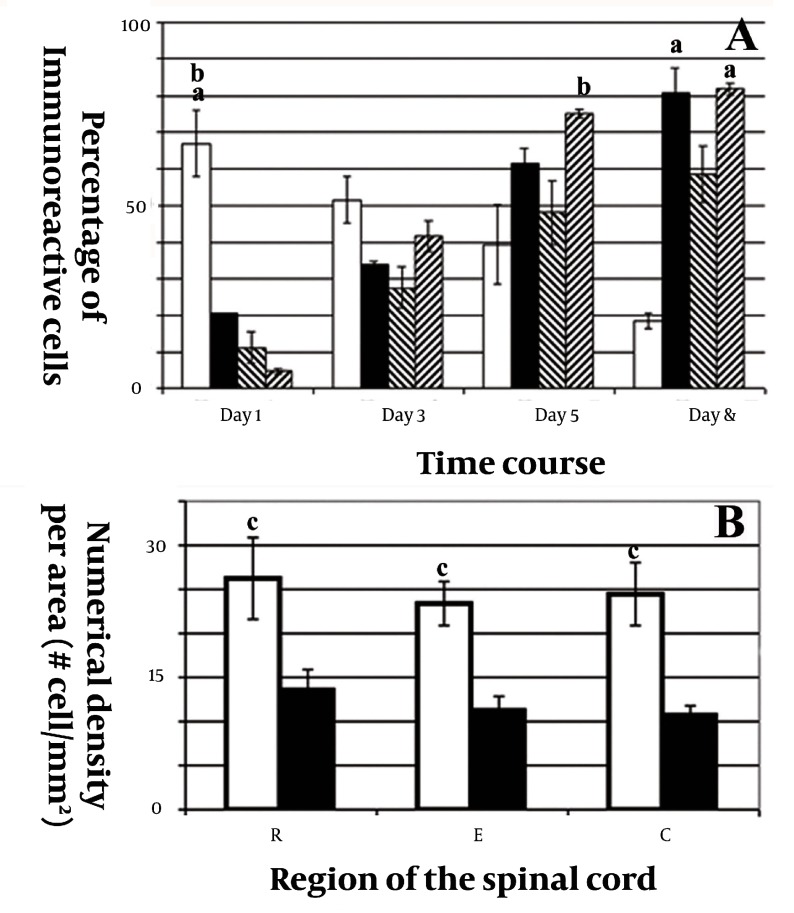
Shows the histograms of the percentages of immunoreactive cells (PIC) to nestin, synaptophysin, NeuN and ChAT (A), and the numerical density per area of the transplanted trans differentiated cholinergic neuron-like cells (B)

**Figure 7:**
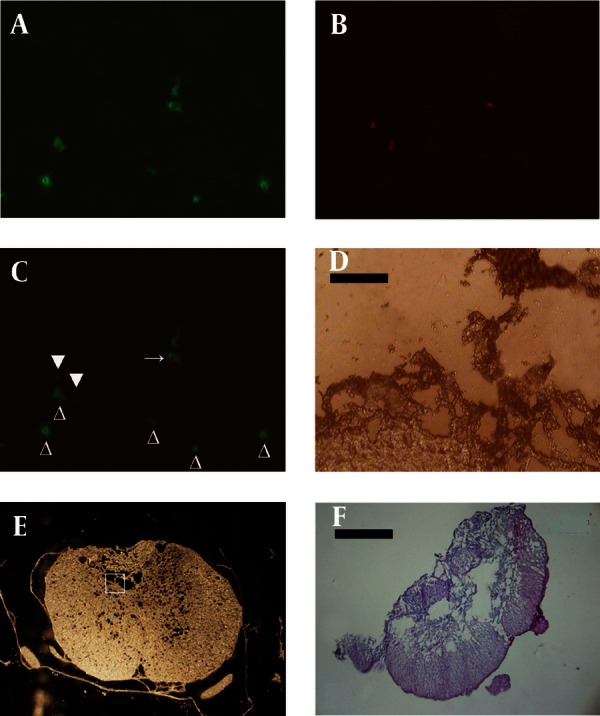
Immunostaining of the induced BMSCs into cholinergic neuron phenotypes labeled with bromodeoxyuridine (BrdU) and transplanted in the rats' contused spinal cord, the animals are sacrificed after 3 months

**Figure 8:**
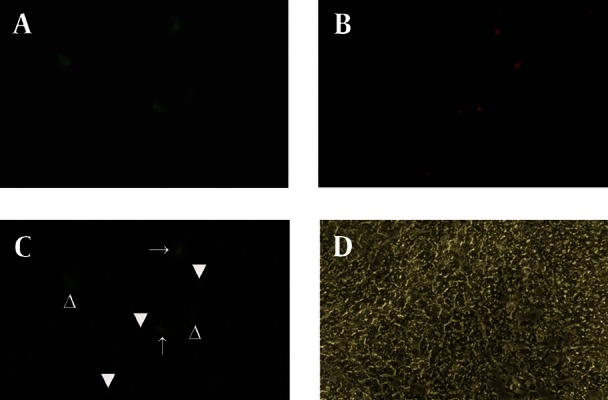
A) Cholinergic neuron phenotypes and endogenousChAT positive cells simultaneously labeled with mouse anti-ChAT monoclonal antibody (primary antibody) and incubated with (secondary antibody) rabbit anti-mouse antibody conjugated with FITC. B) Cholinergic neuron phenotypes labeled with mouse anti-BrdU monoclonal antibody (primary antibody) and incubated with rabbit anti-mouse antibody conjugated with Rhoda mine. C) The images A and B merged showing a double labeled transplanted cell (arrow), empty arrowhead represents a cell immunostained with anti-ChAT antibody which is the host neuron immunoreactive to anti-ChAT antibody, and arrowhead presents a non-cholinergic neuronal transplanted cell. D) A phase contrast image of A, B and C (scale bar = 50 μm)

Figure 1 (A-D) shows the morphology of freshly isolated adherent stromal cells after 24 hours; they were isolated at the 5th passage and pre induced. The BMSCs were evaluated by immunostaining the CD markers, the isolated BMSCs after the 5th passage were immunoreactive to anti-CD44 (Figure 1 E) and were negatively immunostained with anti-CD45 antibodies (Figure 1 F), moreover, they expressed Oct-4 (Figure 2). Figure 3 shows the immunoreactivity of the BMSCs to anti-fibronectin antibody, a marker for BMSCs (Figure 3 A). The trans differentiated BMSCs into cholinergic neuron-like cells (CNL) were labeled with BrdU (Figure 3 F). A comparison of immunoreactivity to neurofilament 68 KD (NF-68) between the pre induced and the induced BMSCs is presented in Figure 3 (C and E); there were more NF-68 immunoreactive cells in the pre induction than the induction stages. Likewise, immuno staining of antigens characterizing mature neurons including neurofilament 200 (NF-200: Figure 3 B and D), synaptophysin (Figure 4 A and C), MAP-2 (Figure 4 B and E) and ChAT (Figure 4-D and F) was done in the pre induced and the induced BMSCs, where the percentage of the immunoreactive cells in NF-200, synaptophysin and ChAT at the induction stage was more than that of the pre induction one, while MAP2 expression was variable. The result of BBB test in the CNL treated group was significantly higher than that of the BMSCs one at 11thday and the 2th, 3th and 4th weeks, while the score was not significant in the other time points (Figure 5 A) and the score of the sham-operated animals were significantly higher than that of the other groups. ANOVA showed that BBB score was significantly higher in the animals treated with CNLs and BMSCs than the untreated animals and those treated with normal saline. Figure 5 (B-E) shows the nonlinear regression by using the Logistic model for BBB score versus time (in days) in the untreated animals, accordingly the curve fitting was done for the normal saline group, the animals treated with BMSCs and those treated with CNLs. Table 1 shows the improvement rates and the improvement indices in the groups. The lowest index was noticed in the NS group, while the highest index was in the CNL group, however, the latter shows higher improvement rate than the BMSCs group. The improvement rate is the “c” coefficient in the logistic model, while the improvement index is the ratio of the improvement rate in any group to that of the sham operated group. Figure 6 A shows the sustained decline in the percentage of nesting immunoreactive cells while the reverse is correct for the values of NeuN, synaptophysin and ChAT. The numerical density of the transplanted BMSCs per area was significantly higher than those of the CNLs (Figure 6 B). The results of the volume densities of the cavitation in the contused spinal cord are presented in Table 2, which shows that the volume density in the C and NS groups is higher than that in the CNL and BMSCs ones. The volume density of the CNL treated group is higher than that of the BMSCs group. Similar results were noticed in the spinal tissue regeneration index. The BrdU labeled cells were injected intraspinally and the animals were sacrificed after 3 months. Figure 7 (A-D) demonstrates the localization of these cells at the dorsal horn from the spinal cord of the animals treated with CNLs. The tissue section was immunostained with mouse anti-BrdU antibody, incubated with rabbit anti-mouse antibody conjugated with Rhoda mine (Figure 7 B), double immunostained with mouse anti-ChAT antibody and incubated with rabbit anti-mouse antibody conjugated with FITC (Figure 7 A). The merged images of ChAT (FITC) and BrdU (Rhoda mine) double labeled cells are shown in Figure 7 C, while the phase contrast photomicrograph can be seen in Figure 7 D. Figure 7 E shows the regeneration and the comparison with the negative control (Figure 7 F). Double immunostained CNLs were localized at the ventral horn (double stained for BrdU and ChAT: Figure 8).

**Table 1:** Improvement Rate and the Improvement Index in the Sham Operated

Group	Improvement rate	Improvement index, %
**Sham**	0.18434158	100
**Contusion**	0.11602318	62.94
**Normal saline**	0.10018388	54.35
**BMSCs**	0.1085612	58.89
**Cholinergic**	0.1710525	92.79

**Table 2:** Morphometric Data Using the Mean Volume Density of the Cavity

Site of cavitation	SO a	C1 b, Mean ± SD	C2 b, Mean ± SD	E1 c, Mean ± SD	E2, Mean ± SD
** (V̅ v_cavity (R)_) d**	0	0.16 ± 0.02	0.169 ± 0.02	0.073 ± 0.013	0.072 ± 0.013
** (V̅ v_cavity (E)_) e**	0	0.33 ± 0.05	0.296 ± 0.03	0.16 ± 0.012	0.15 ± 0.02
** (V̅ v_cavity (R)_) f**	0	0.169 ± 0.01	0.146 ± 0.02	0.066 ± 0.01	0.06 ± 0.014
** STRI (R) g**	-	-	-	156.8	155
** STRI (E) h**	-	-	-	151.52	154.55
** STRI (C) i**	-	-	-	160.95	164.5

^a^means significantly lower than all other groups

^b^means significantly higher than the experimental groups

^c^means no significant difference with other than the other experimental group

^d^the mean of the volume density of the spinal cavitation at the rostral region of the spinal cord of the epicenter injury site

^e^the mean of the volume density of the spinal cavitation at the epicenter site of the spinal cord.

^f^the mean of the volume density of the spinal cavitation at the caudal region of the spinal cord of the epicenter injury site

^g^the spinal tissue regeneration index in the contused rats at the rostral region of the injury epicenter site

^h^the spinal tissue regeneration index in the contused rats at the injury epicenter site

^i^the spinal tissue regeneration index in the contused rats at the caudal region of the injury epicenter site

## 5. Discussion

The percentage of the cholinergic neuron phenotypes pre induced with β-mercaptoethanol and induced with NGF was consistent with a previous investigation (25). From the in vitro study, the time point for their transplanting should be decided from the time course, the selection of this time point was based on two criteria: the differentiation property and the integrity of the transplanted cells. The differentiation criterion is the highest number of the induced cells immunoreactive to the neuroprogenitor cell marker (nestin). The reason for selecting the neuroprogenitor cell is its being the best choice for replacement therapy for neurodegenerative diseases (28). Moreover, the induced undifferentiated stem cells into neural phenotypes could engraft, survive the transplantation and promote axonal growth (29). The neural precursor cells have the potential to differentiate into all neural cell types in the injured spinal cord (30) . However, Cao et al. revealed that inducing neural stem cells could result in their differentiation into glial lineage with few neuron-like cells (31). Therefore, the best option is to select the neuro progenitor cells committed to cholinergic phenotypes, where the percentages of immunoreactive cells to nestin and ChAT were high at 3rd and 5th days (25). The other criterion is the integrity of the transplanted cells, the selected neuroprogenitor cells at 3rd day, according to the above criterion, had less synaptophysin expression than those at 5th day. The synaptophysin involved in the synaptic contact at the axons or other extensions (23), harvesting the neuron-like cells in order to transplant them, can cause axotomy or damage to other extensions, which are essentially injured neurons with less chance of survival as transplants (25). The transplantation of these cells in the injured spinal cord resulted in improvement of BBB score compared with the untreated animals. The trend of improvement with significantly higher BBB score was noticed in the cholinergic phenotype treated group more than in those treated with BMSCs at the first two weeks, while in the following weeks (3-12 weeks), the data showed no significant differences. However, the numerical density per area in the cholinergic phenotype treated group at the end of the experiment was about 50% of that of the group treated with BMSCs. The possible explanation for the reduction in the number of cholinergic transplants is that they were unable to survive the transplantation. The induction protocol of the BMSCs into cholinergic phenotypes by NGF, used in this study, could result in their dependency on NGF with conditioning of survival on the presence of NGF in the culture medium (32). Their transplantation in the injured spinal cord was characterized by lack of trophic factors. Moser et al. reported that NGF could block cholinergic neuron death induced by neurotoxin (33), also Ahlemeyer et al. documented the protective effect of NGF against apoptosis inducers (34). The most important point about the transplantation of the cholinergic neuron-like cells into the spinal cord is that the in vivo new microenvironment may not provide sufficient NGF to these cells, thus they start to die depending on their maturity (35) and the possible reason for cholinergic neuron-like cells death is deprivation of NGF (36). The experiment was terminated as the improvement reached a plateau (12 weeks) (37). The non-linear regression of BBB score using the logistic model showed that initially the rate of improvement was higher in the cholinergic phenotype treated group than those treated with the BMSCs, which may indicate that the cholinergic phenotypes could improve the functional activity better than the BMSCs. The possible reason for the lowest improvement index in the normal saline treated group is that the contusive rats were subjected to reoperation at one week old surgical wound, where the wound was reopened and the spinal cord was injected with the vehicle and then closed again, these procedures may traumatized the already contused spinal cord. Similar procedures were carried out in the other groups such as the BMSCs and the cholinergic phenotype treated ones. This may explain the low result of the improvement index in the BMSCs animals compared to those subjected to the contusion injury without reoperation. The explanation is that the improvement index was calculated by taking the ratio of the improvement rate of a given group to that of the sham operated group, the second trauma resulted from reopening the operation site and injecting the BMSCs, which caused the decline in the rate of improvement. However, the improvement rate in those animals injected with normal saline was lower than those injected with the BMSCs. The improvement of the contused spinal cords using cholinergic transplant is consist with the results of Gao et al. (22), who reported that cholinergic neurons differentiated from neural stem cells were able to innervate the target muscle and cause motor function improvement, while other investigators reported similar results from transplanting BMSCs in the injured spinal cord (38). Also Dezawa et al. (39) revealed that BMSCs could successfully integrate the injured spinal cord and cause behavioral improvement. Clinical trials by other investigators reported improvement of the traumatized spinal cord (40, 41). The transplantation of neuronal lineages differentiated from bone marrow could promote the recovery of mice with spinal cord injury with significant improvement of the motor function; it may explain the higher improvement index compared with that of the BMSCs (42). Also, Tarasenko et al. revealed that the pre differentiation stage of the transplanted human neural stem cells was a determining factor in the outcome of the functional improvement of the contused rats (21). Table 1 shows the improvement rate and the improvement index in the sham operated (sham), the rats with contusion spinal injury without any treatment (contusion), the rats with contusion spinal injury injected with normal saline (normal saline), the rats with contusion spinal injury injected with bone marrow stromal cells (BMSCs) and the rats with contusion spinal injury injected with trans differentiated cholinergic neuron-like cells (cholinergic).

**Figure 1:** A) Freshly prepared BMSCs (scale bar = 500 μm); B) BMSCs 24 hours after culturing shows adherent cells (scale bar = 500 μm); C) BMSCs after the 5th passage shows homogenous adherent spindle-shape cells (scale bar = 100 μm); D) Pre induced BMSCs with β-mercaptoethanol after 12 hours, arrow indicates neuron-like cells with multipolar extensions, arrowhead represents an axon-like extension (scale bar = 200 μm); E) Immunostaining of BMSCs with anti-CD44 monoclonal antibody (primary antibody) then labeled with (secondary) FITC conjugated rabbit anti-mouse antibody. The cells are counterstained with ethidium bromide; F) Immunostaining of BMSCs with anti-CD45 monoclonal antibody (primary antibody) then labeled with (secondary) FITC conjugated rabbit anti-mouse antibody. The cells are counterstained with ethidium bromide; H) represents the phase contrast of G (scale bar = 37.5 μm: all).

**Figure 2:** The BMSCs show a sharp band indicating their stemness, B2M (β2 microglobulin) is internal control

**Figure 3:** A) Immunostaining of BMSCs with anti-fibronectin monoclonal antibody (primary antibody), then labeled with (secondary antibody) FITC conjugated rabbit anti-mouse antibody, the cells are counterstained with ethidium bromide; B) Immunostaining of the pre induced BMSCs with anti-NF-200 monoclonal antibody, then labeled with (secondary antibody) FITC conjugated rabbit anti-mouse antibody, the cells are counterstained with ethidium bromide; C) immunostaining of the preinduced BMSCs with anti-NF-68 monoclonal antibody, then labeled with (secondary antibody) FITC conjugated rabbit anti-mouse antibody, the cells are counterstained with ethidium bromide; D) immunostaining of the induced BMSCs with anti-NF-200 monoclonal antibody, then labeled with (secondary antibody) FITC conjugated rabbit anti-mouse antibody, the cells are counterstained with ethidium bromide; F) induced BMSCs labeled with anti-bromodeoxyuridine (BrdU) monoclonal antibody, then labeled with (secondary antibody) FITC conjugated rabbit anti-mouse antibody. (Scale bar = 15 μm: all).

**Figure 4:** A) Immunostaining of the pre induced BMSCs with anti-synaptophysin monoclonal antibody, then labeled with (secondary antibody) FITC conjugated rabbit anti-mouse antibody, the cells are counterstained with ethidium bromide. B) Immunostaining of the induced BMSCs with anti-MAP2 monoclonal antibody, then labeled with (secondary antibody) FITC conjugated rabbit anti-mouse antibody, the cells are counterstained with ethidium bromide. C) Immunostaining of the induced BMSCs with anti-synaptophysin monoclonal antibody, then labeled with (secondary antibody) FITC conjugated rabbit anti-mouse antibody, the cells are counterstained with ethidium bromide. D) Immunostaining of the pre induced BMSCs with anti-ChAT monoclonal antibody, then labeled with (secondary antibody) FITC conjugated rabbit anti-mouse antibody, the cells are counterstained with ethidium bromide. E) Immunostaining of the pre induced BMSCs with anti-MAP2 monoclonal antibody, then labeled with (secondary antibody) FITC conjugated rabbit anti-mouse antibody, the cells are counterstained with ethidium bromide. F) Immunostaining of the pre induced BMSCs with anti-ChAT monoclonal antibody, then labeled with (secondary antibody) FITC conjugated rabbit anti-mouse antibody, the cells are counterstained with ethidium bromide (scale bar = 75 μm: all).

**Figure 5:** B-E represents the logistic curve fitting of BBB test data. B) Represents the logistic model in the sham operated animals [y=21.008/1+0.2c-0.184x]; standard error = 0.040270352, correlation coefficient = 0.93259736. C) Represents the logistic model in the negative control group injected with normal saline[y=11.64/1+4.86c- 0.1002x]; standard error = 0.46430323, correlation coefficient = 0.99109102. D) Represents the logistic model in the animals transplanted with BMSCs [y=14.2/1+5.49c- 0.109x]; standard error = 0.71573168, correlation coefficient = 0.98984847. E) Represents the logistic model in the sham operated animals transplanted with trans differentiated BMSCs into cholinergic neuron phenotypes [y=13.83/1+c6.98-0.171x]; standard error = 0.74102311, correlation coefficient = 0.98836318. “a” means that the animal group treated with cholinergic neuron-like cells is significantly higher than the other group at that time point.

**Figure 6:** Shows a histogram of the percentages of immunoreactive cells to nestin (white solid column), synaptophysin (black solid column), NeuN (down cross-hatched column) and ChAT (up cross-hatched column) in the preinduction phase (1st day) and induction phase at 3rd, 5th and 7th daysfrom the start of the differentiation protocol. B) Shows a histogram of the numerical density per area, which represents the number of the survived bone marrow stromal cells (white solid column) and the trans differentiated cholinergic neuron phenotypes (black solid column), after 12 weeks of transplantation surgery, at the epicenter the of impact site of the spinal cord (E), the rostral (R) and the caudal (C) of the site. “a” means that the PIC to this marker is significantly higher at this time point than the other time points. “b” means that the PIC to this marker is significantly higher at this time point than the other markers. “c” means the numerical density of the transplanted BMSCs per area, which is significantly higher than those of CNLs.

**Figure 7:** Cholinergic neuron phenotypes and endogenous ChAT positive cells simultaneously labeled with mouse anti-ChAT monoclonal antibody (primary antibody) and incubated with rabbit anti-mouse antibody conjugated with FITC. B) Cholinergic neuron phenotypes labeled with mouse anti-BrdU monoclonal antibody (primary antibody) and incubated with (secondary antibody) rabbit anti-mouse antibody conjugated with Rhoda mine. C) The images A and B merged showing a double labeled transplanted cell (arrow), empty arrowhead represents a cell immunostained with anti-ChAT antibody which is the host neuron immunoreactive to anti-ChAT antibody; arrowhead represents a non-cholinergic neuronal transplanted cell. D) A phase contrast image of A, B and C (X 400), this represents the region of the square in E (scale bar = 25 μm). F) A tissue section stained with H&E from a spinal cord of a rat used as negative control, it is injected with normal saline and shows large cavities in the contused spinal cord (scale bar = 5000 μm).
